# Supplementary figures and images for: A comparison of rumen microbial profiles in dairy cows as retrieved by 454 Roche and Ion Torrent (PGM) sequencing platforms
Source: PeerJ. 2016 Feb 4;4:e1599. doi: 10.7717/peerj.1599 (PMC4748696; doi:10.7717/peerj.1599)

[Paraprevotellaceae]

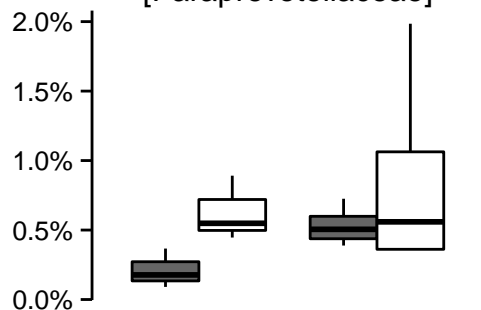

Bacteroidales

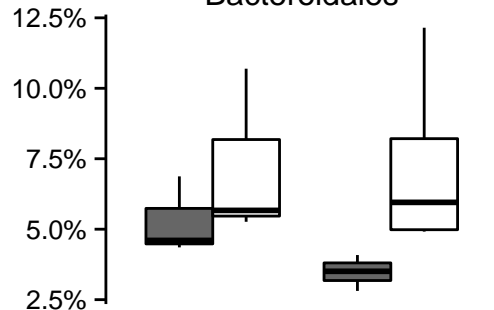

CF231

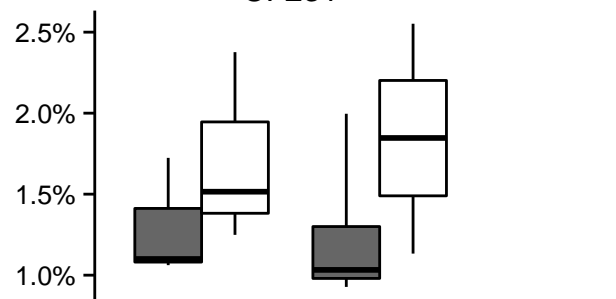

Prevotella

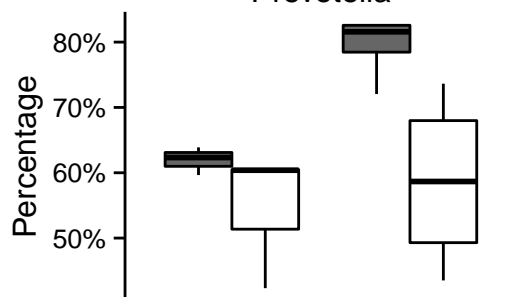

Prevotellaceae

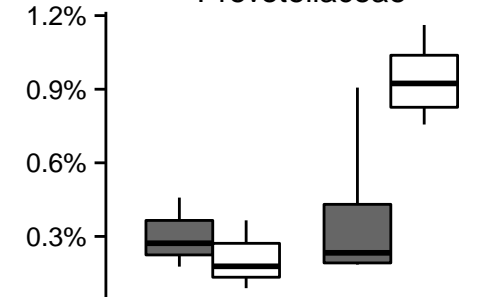

RF16

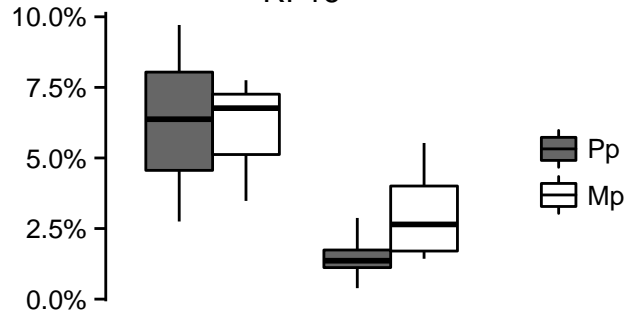

S24-7

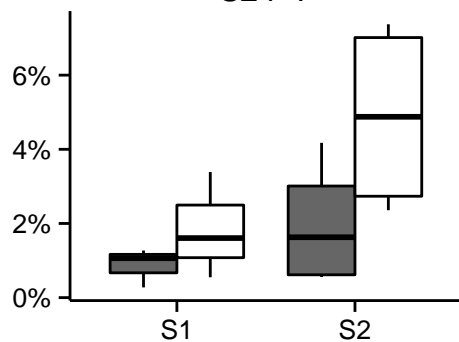

YRC22

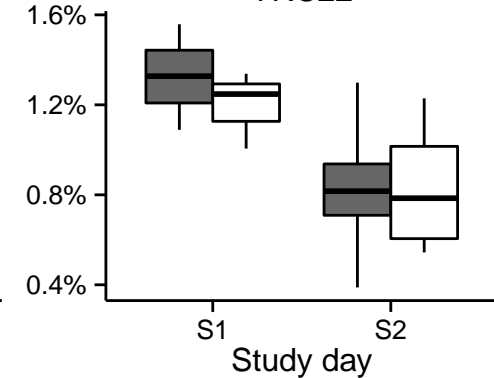

Pp  
Mp

Supplement: Figure S1 [file peerj-04-1599-s003.pdf]

[Paraprevotellaceae]

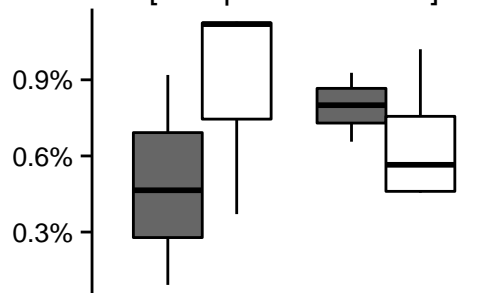

Bacteroidales

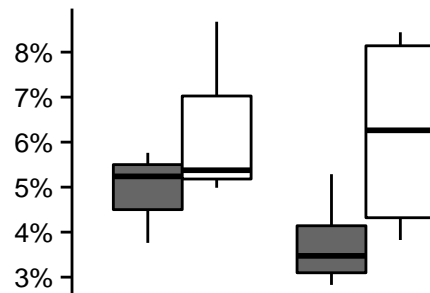

BF311

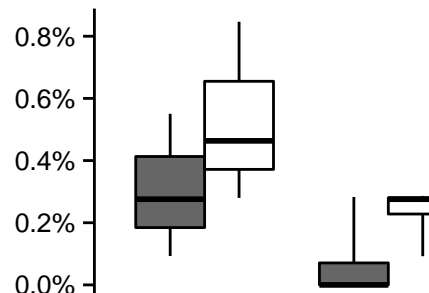

CF231

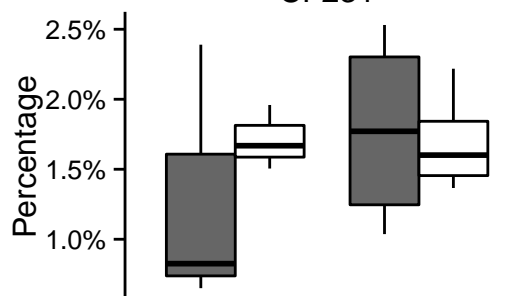

Prevotella

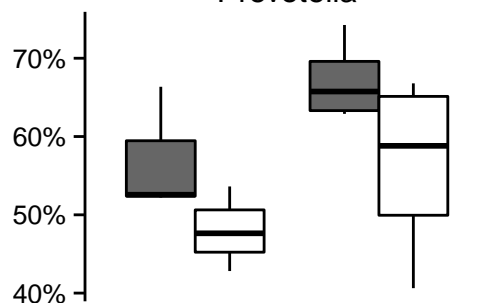

Prevotellaceae

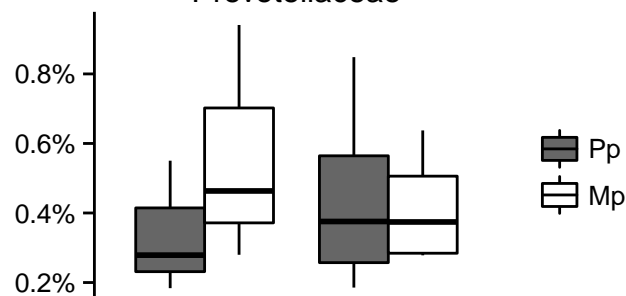

RF16

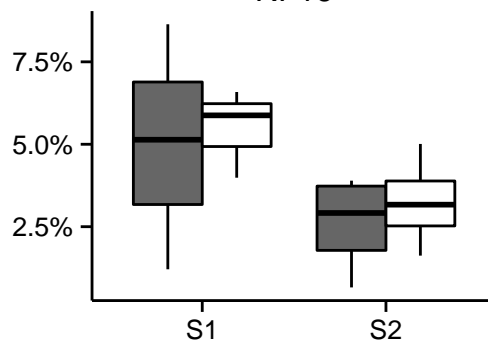

S24-7

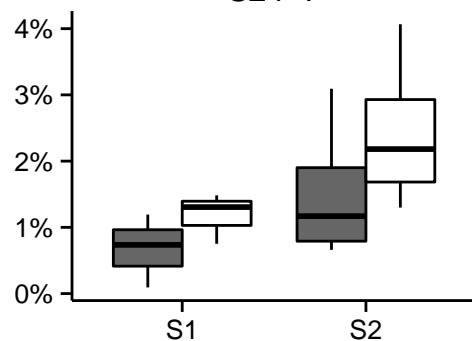

YRC22

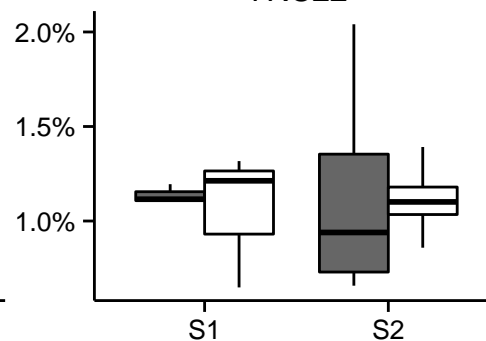

Pp  
Mp

Supplement: Figure S2 [file peerj-04-1599-s004.pdf]

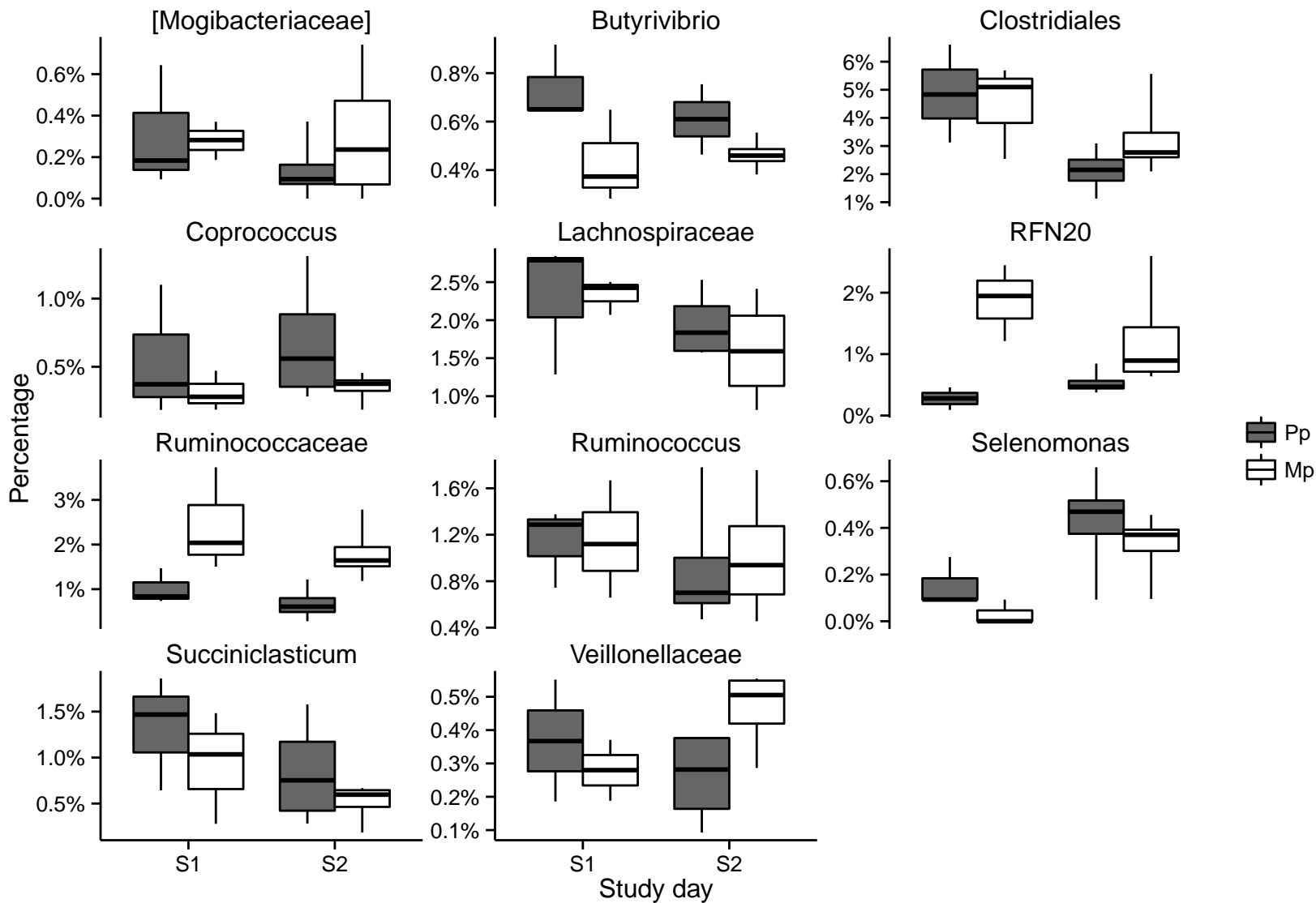

Supplement: Figure S4 [file peerj-04-1599-s007.pdf]
