# Supplementary material for: A comparison of rumen microbial profiles in dairy cows as retrieved by 454 Roche and Ion Torrent (PGM) sequencing platforms
Source: PeerJ. 2016 Feb 4;4:e1599. doi: 10.7717/peerj.1599 (PMC4748696; doi:10.7717/peerj.1599)
Supplement: Figure S3 [file peerj-04-1599-s006.pdf]

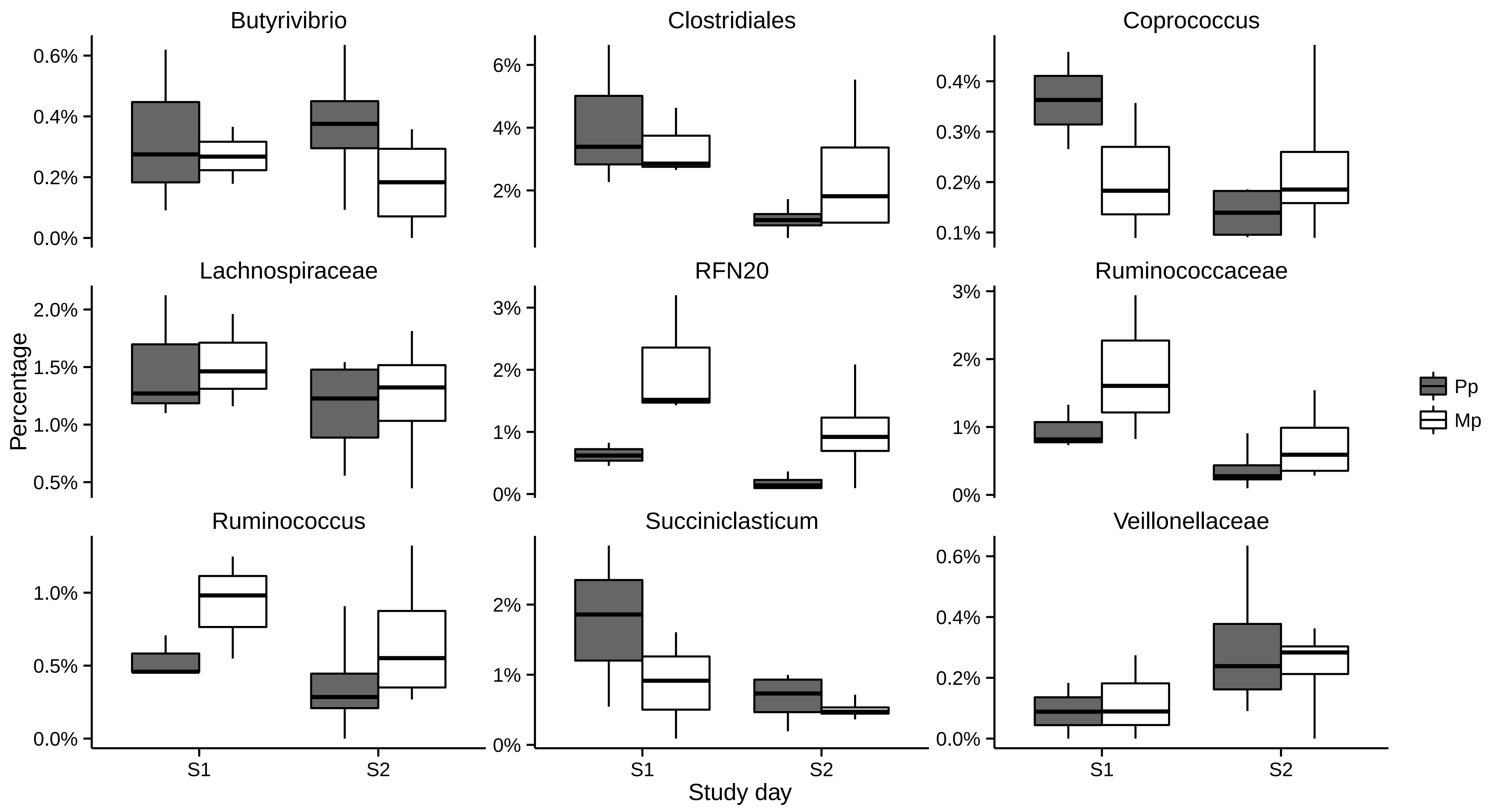

Butyrivibrio
Clostridiales
Coprococcus
Lachnospiraceae
RFN20
Ruminococcaceae
Ruminococcus
Succiniclasticum
Veillonellaceae
0.6%
0.4%
0.2%
0.0%
6%
4%
2%
0.4%
0.3%
0.2%
0.1%
2.0%
1.5%
1.0%
0.5%
3%
2%
1%
0%
1.0%
0.5%
0.0%
Percentage
S1
S2
Study day
Pp
Mp
